# Revision of the plant bug genus *Xenocylapidius* (Hemiptera, Heteroptera, Miridae, Cylapinae), with descriptions of five new species from Australia and New Caledonia

**DOI:** 10.3897/zookeys.459.8015

**Published:** 2014-12-02

**Authors:** Andrzej Wolski, Jacek Gorczyca

**Affiliations:** 1 Department of Biosystematics, Opole University, Oleska 22, 45–052 Opole, Poland; 2 Department of Zoology, University of Silesia, Bankowa 9, 40–007 Katowice, Poland

**Keywords:** Heteroptera, Miridae, Cylapinae, *Xenocylapidius*, new species, keys, Australian Region, Australia, New Caledonia

## Abstract

The genus *Xenocylapidius* Gorczyca, 1997 is revised. Five new species: *Xenocylapidius acutipennis* **sp. n.**, *Xenocylapidius ater* **sp. n.**, *Xenocylapidius bimaculatus* **sp. n.**, *Xenocylapidius gemellus* **sp. n.**, and *Xenocylapidius rolandi* **sp. n.** are described from Australia and New Caledonia. Illustrations of the male genitalia, color photographs of dorsal and lateral views of the adults of all species, and key to species of the genus *Xenocylapidius* are provided.

## Introduction

With 75 species included in 28 genera (Schuh 2002–2013; Gorczyca 2006; Wolski 2012; Wolski and Gorczyca 2014) the Cylapinae in the Australian Region remain one of the most poorly known mirid subfamilies. Most of our knowledge about the Australian representatives of the Cylapinae is based on Carvalho and Lorenzato (1978), who reviewed the Papuan cylapines, Cassis et al. (2003), and Moulds and Cassis (2006), who provided revisionary treatments of the Australian species of Vaniini and the genus *Peritropis* Uhler, 1981, respectively.

The genus *Xenocylapidius* was described by Gorczyca (1997) to accommodate a new species *X. tamasi.* Subsequently, Gorczyca (1999) added two species – *Xenocylapidius australis* and *Xenocylapidius gressitti*, provided a redescription of the genus and type species and a key to species. Gorczyca (2006) transferred *Rhinomiridius bioculatus* Girault to *Xenocylapidius* and synonymized *Xenocylapidius australis* with this species.

In this contribution, we revise the genus *Xenocylapidius* and describe five new species. All previously known species are diagnosed, and identification key to species is provided.

## Materials and methods

Observations were made using an Olympus SZX12 stereomicroscope and an Olympus BX50 optical microscope. Color pictures of the adults (Figs 1–15) were taken with an ALTRA 20 digital camera. Additional information on the pictured specimens is given in the species treatments.

Measurements were taken using an eyepiece (ocular) micrometer; all measurements are given in millimeters. The total body length is defined by the length from the apex of the clypeus to the posterior margin of the membrane, and the body width by the length between the lateral margins of the hemelytra at their widest point. Lengths and widths of the head are defined as follows: length, from the apex of the clypeus to the occipital carina; width, between the outer margins of each eye; diameter of eye, between the outer and inner margin of eye; length of the antennal and labial segments, between the base and apex. Lengths and widths of the pronotum are defined as follows: length, measured between the anterior and posterior margins; width of the anterior margin, between anterior angles; length of lateral margin, between the anterior and humeral angles; width of the posterior margin, between the humeral angles.

Dissections of male genitalia were done according to Kerzhner and Konstantinov (1999). The terminology of the male genitalic structures follows Konstantinov (2003) and Cassis (2008). The following additional terms for the elements of the endosoma are used in this paper:

AR – apical ring – apical portion of basal sac, composed of tiny spiculi and denticles, not forming a fully closed ring;

BP – basal fig – irregularly shaped, sclerotized figd situated at base of the endosoma;

BSC – basal sac – sclerotized sac situated at base of the endosoma, almost entirely embracing sclerotized portion of ductus seminis inside the endosoma (DSS);

DLS – dextrolateral sclerite – situated on the dextrolateral portion of the apical part of the endosoma;

DSS – sclerotized portion of ductus seminis inside endosoma;

MS – medial sclerite – situated at middle of the endosoma, with base localized near apex of DSS;

SLS – sinistrolateral sclerite – situated on the sinistrolateral portion of the apical part of the endosoma;

SP1, SP2, and SP3 – endosomal spiculi – bundles of spiculi situated basally, medially, and apically on the endosoma.

The material examined includes specimens borrowed from the institutions listed below:

BPBM Department of Entomology Collection, Bernice P. Bishop Museum, Honolulu, Hawaii, USA;

HNHM Hungarian Natural History Museum, Budapest, Hungary;

MNHN Museum National d’Histoire Naturelle, Paris, France;

NHRS Naturhistoriska Riksmuseet, Stockholm, Sweden;

US Department of Zoology, University of Silesia, Katowice, Poland;

USNM National Museum of Natural History, Smithsonian Institution, Washington, D.C., USA;

ZSM Zoologische Staatssammlung München, Munich, Germany.

## Taxonomy

### Xenocylapidius

Taxon classificationAnimaliaHemipteraMiridae

Gorczyca

Xenocylapidius : Gorczyca 1997: 179, 183 (sp. n.), 1999: 16 (key to species), 2000: 49 (list), 2006: 70 (catalog); Chérot and Gorczyca 1999: 217 (note); Carpintero and Chérot 2014: 62 (note).

#### Diagnosis.

Recognized by the following combination of characters: labial segment II subdivided medially or subapically; lateral margin of pronotum somewhat elevated; scent gland efferent system broad, occupying entire ventral margin of metepisternum; endosoma with a characteristic sclerotized basal sac with a relatively broad, ringlike structure apically (AR = apical ring) that is composed of numerous denticles and spiculi (Figs 16–17, 21–22, 26–27, 32–33, 37–38, 42–43); left paramere with a long, protruding sensory lobe (SL) (Figs 18–19, 23–24, 28–29, 34–35, 39–40, 44–45).

#### Redescription.

**Figures 1–8.**
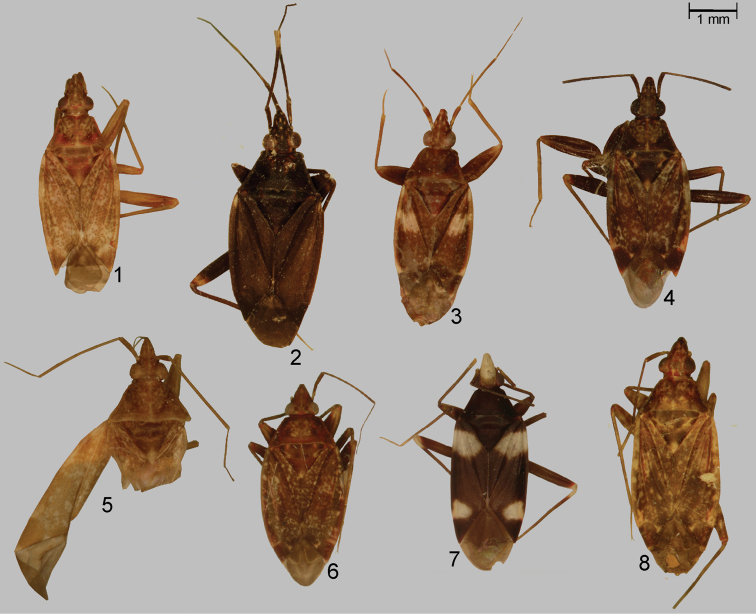
Dorsal habitus color photographs of *Xenocylapidius* spp.: **1** *Xenocylapidius acutipennis* (holotype) **2** *Xenocylapidius ater* (holotype) **3** *Xenocylapidius bimaculatus* (holotype) **4** *Xenocylapidius bioculatus* (♀: Australia N. S. W., Manly nr Sydney, North Head 16–21.2., D. Shcherbakov 1997) **5** *Xenocylapidius gemellus* (holotype) **6** *Xenocylapidius gressitti* (holotype) **7** *Xenocylapidius rolandi* (holotype) **8** *Xenocylapidius tamasi* (♀: New Caledonia, Foret di Thi, 29.X.–1.XI.1967).

**Figures 9–15.**
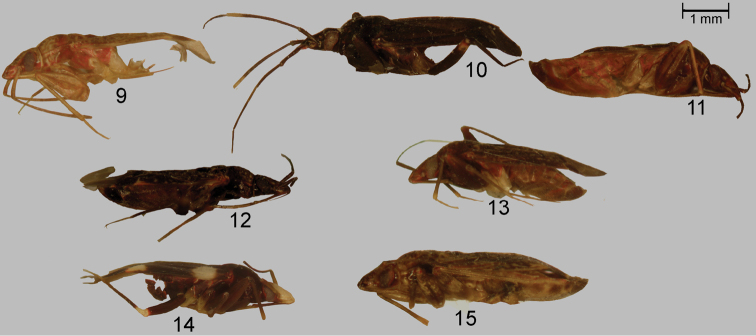
Color photographs of *Xenocylapidius* spp., lateral views: **9** *Xenocylapidius acutipennis* (holotype) **10** *Xenocylapidius ater* (holotype); 11. *Xenocylapidius bimaculatus* (holotype) **12** *Xenocylapidius bioculatus* (♀: Australia N. S. W., Manly nr Sydney, North Head 16–21.2., D. Shcherbakov 1997) **13** *Xenocylapidius gressitti* (holotype) **14** *Xenocylapidius rolandi* (holotype) **15** *Xenocylapidius tamasi* (♀: New Caledonia, Foret di Thi, 29.X.–1.XI.1967).

**STRUCTURE, TEXTURE, AND VESTITURE** (Figs 1–15). Macropterous, elongate oval. ***Head*.** Elongate horizontally, conical; antennal segment I gradually thickened toward apex, covered with sparse, short, adpressed setae and sometimes covered with a few bristlelike, protruding setae apically; segment II weakly broadened toward apex, covered with moderately dense, semirecumbent setae and sometimes with sparse, bristlelike, protruding setae on apical half; segments III and IV thin, with diameter about twice as thin as diameter of segment II, mixed with long, moderately dense, semirecumbent setae and with a few, bristlelike, protruding setae; labium thin, reaching medial part of abdomen or beyond; segment I subdivided near medial part, extending beyond base of head to anterior edge of xyphus; segment II subdivided subapically. ***Thorax*.** *Pronotum*. Trapezoidal; collar present, thin; humeral angle usually furnished with single, bristlelike, rather long, protruding seta; calli moderately convex, broad, occupying anterior two thirds of pronotum; lateral margin usually strongly carinate and somewhat elevated, rarely weakly carinate and not elevated; posterior margin arcuate. *Mesoscutum and scutellum*. Mesoscutum well exposed; scutellum flattened or weakly convex. *Thoracic pleura*. Proepisternum and proepimeron shiny; remaining pleura matte; scent gland efferent system broad, occupying entire ventral margin of metepisternum. *Hemelytron*. Usually covered with very short, relatively dense, adpressed, black setae, rarely with sparse, relatively long, protruding setae; membrane with major cell nearly rectangular, minor cell clearly present. *Legs*. Relatively long; profemur usually with several protruding, thick, relatively long setae on inner surface; tarsus bisegmented; tarsomere II subdivided medially; pretarsal claw toothed subapically.

**Figures 16–25.**
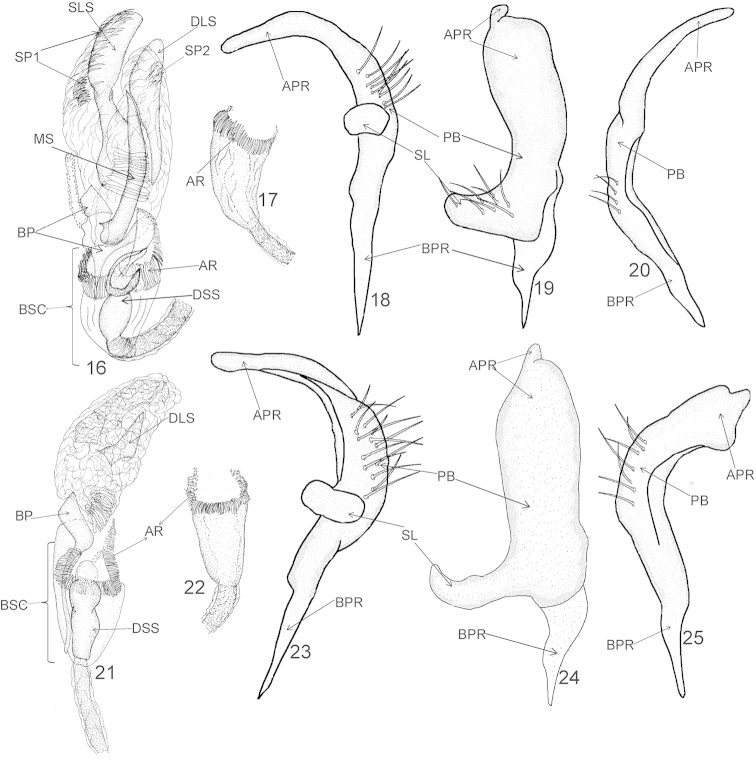
Male genitalia of *Xenocylapidius acutipennis* (**16–20**) and *Xenocylapidius ater* (**21–25**): **16, 21** Endosoma (dorsal view) **17, 22** Basal sac of endosoma (ventral view) **18, 23** Left paramere (left lateral view) **19, 24** Left paramere (dorsal view) **20, 25** Right paramere (right lateral view). APR = apical process of paramere; AR = apical ring of endosomal basal sac; BP = basal fig; BPR = basal process of paramere; BSC = basal sac; DLS = dextrolateral sclerite; DSS = sclerotized portion of ductus seminis inside endosoma; MS = medial sclerite; PB = paramere body; SL = sensory lobe; SLS = sinistrolateral sclerite; SP1 and SP2 = endosomal spiculi.

**Figures 26–30.**
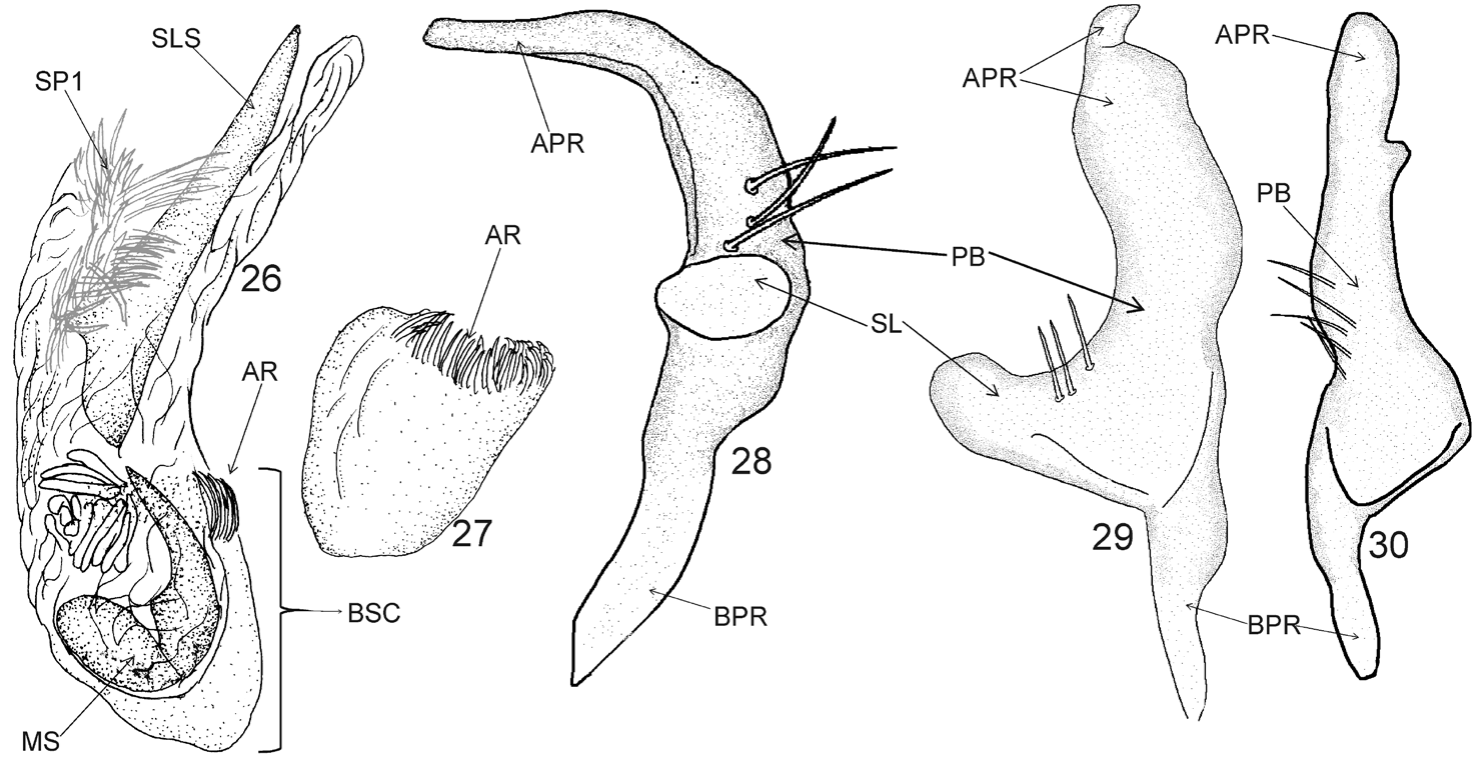
Male genitalia of *Xenocylapidius bimaculatus*: **26** Endosoma (dorsal view) **27** Basal sac of endosoma (ventral view) **28** Left paramere (left lateral view) **29** Left paramere (dorsal view) **30** Right paramere (right lateral view). APR = apical process of paramere; AR = apical ring of endosomal basal sac; BPR = basal process of paramere; BSC = basal sac; DSS = sclerotized portion of ductus seminis inside endosoma; PB = paramere body; SL = sensory lobe; SLS = sinistrolateral sclerite; SP1 = endosomal spiculi.

***Male genitalia*.** *Aedeagus* (Figs 16–17, 21–22, 26–27, 32–33, 37–38, 42–43). Ductus seminis thin, with an outer wall fine and membranous; base of endosoma with a sclerotized sac (BSC), occupying one third to almost half of endosoma, enveloping sclerotized part of ductus seminis inside endosoma (DSS), with a large, not fully closed apical ring (AR) composed of tiny spiculi or/and denticles; secondary gonopore distinct; endosoma usually with 1–3 bundles of distinct spicules (SP1, SP2, and SP3); base of endosoma sometimes with an irregular, sclerotized fig (BP = basal fig); medial portion of endosoma often with a large sclerite (MS = medial sclerite); apical portion of endosoma with 1-2 large sclerites (dextrolateral sclerite = DLS and a sinistrolateral sclerite SLS). *Left paramere* (Figs 18–19, 23–24, 28–29, 34–35, 39–40, 44–45). Apical process: dorsal view: extreme apex strongly narrowed, usually rounded and weakly curved; paramere body: dorsal surface with bundle of thick, protruding setae; sensory lobe: convex and stout.

#### Remarks.

*Xenocylapidius* is differentiated from other genera of Cylapinae primarily by the presence of the characteristic sclerotized sac at the base of endosoma (BS = basal sac) with the apical portion composed of numerous denticles and spiculi (AR = apical ring) surrounding well developed sclerotized part of ductus seminis inside endosoma (DSS) (Figs 16–17, 21–22, 26–27, 32–33, 37–38, 42–43) and by the large, stout sensory lobe (SL) of the left paramere (Figs 18–19, 23–24, 28–29, 34–35, 39–40, 44–45). In other Cylapinae the endosoma is usually furnished with more or less developed sclerotized part of the ductus seminis (DSS) (e.g. Carvalho and Fontes 1968; Carvalho and Lorenzato 1978; Cassis et al. 2003; Wolski 2010, 2013; Wolski and Henry 2012, Wolski and Gorczyca 2012, 2014) but it never is embraced by the basal sac (BS) as in *Xenocylapidius*.

*Xenocylapidius* is superficially similar to *Peritropis* Uhler, primarily in sharing elevated lateral margins of pronotum but can be easily distinguished by the shape of the male genitalia.

#### Key to species of *Xenocylapidius*

**Table:** 

1	Hemelytron with mottled, brown to blackish and yellow to dirty yellow coloration (Figs 1, 4, 5, 6, 8)	**2**
–	Hemelytron uniformly blackish (Fig. 2) or blackish or chocolate with a white patch near base of corium and embolium (Figs 3, 7), color never mottled	**6**
2	Metafemur brown to dark brown with large, yellow patches (Fig. 8); endosoma with basal sac entirely covered with small denticles (Fig. 43)	***Xenocylapidius tamasi* Gorczyca**
–	Metafemur uniformly dirty yellow to black (Figs 1, 4, 6); endosoma with basal sac without small denticles posteriorly (*Xenocylapidius acutipennis* and *Xenocylapidius gemellus*) (Figs 17, 33)	**3**
3	Apical half of antennal segment II mixed with dense, fine, semirecumbent setae and sparse, protruding, bristlelike setae	***Xenocylapidius bioculatus* (Girault)**
–	Apical half of antennal segment II with only fine, semirecumbent setae, without sparse, protruding, bristlelike setae	**4**
4	Pronotal collar indistinct; yellow mottling on hemelytron composed of relatively small patches and spots (Fig. 6)	***Xenocylapidius gressitti* Gorczyca**
–	Pronotal collar well developed; yellow mottling on hemelytron composed of large patches (Figs 1, 5)	**5**
5	Antennal segment II dark brown (Fig. 9); endosoma with two apical sclerites (DLS and SLS); medial sclerite (MS) long, weakly curved, tapering toward apex, sharply pointed (Fig. 16)	***Xenocylapidius acutipennis* Wolski & Gorczyca sp. n.**
–	Antennal segment II brownish yellow; endosoma with only one apical sclerite (SLS); medial sclerite (MS) with basal one third nearly rounded, apical two thirds tapering toward apex, sharply pointed apically (Fig. 32)	***Xenocylapidius gemellus* Wolski & Gorczyca sp. n.**
6	Hemelytron entirely black (Fig. 2)	***Xenocylapidius ater* Wolski & Gorczyca sp. n.**
–	Hemelytron chocolate brown or black, with a large, white patch near base of corium (Figs 3, 7)	**7**
7	Hemelytron chocolate brown with a large, white patch near base of corium and with a small white patch on embolium apically (Fig. 3)	***Xenocylapidius bimaculatus* Wolski & Gorczyca sp. n.**
–	Hemelytron black with a large, white patch near base of corium and with a large, white patch on apex of embolium, apicolateral surface of corium, and medial portion of inner margin of cuneus (Fig. 7)	***Xenocylapidius rolandi* Wolski & Gorczyca sp. n.**

### Xenocylapidius acutipennis

Taxon classificationAnimaliaHemipteraMiridae

Wolski & Gorczyca sp. n.

http://zoobank.org/C6849117-A190-43E2-8017-34C5A33D03F4

Figures 1 , 9 , 16 –20 , 31

#### Diagnosis.

Recognized by the dorsum mottled with brownish yellow (Fig. 1); the dark brown antennal segment II; the endosoma with two bundles of spiculi (SP1 and SP2); the medial sclerite (MS) long, weakly curved, tapering toward apex, sharply pointed; the sinistrolateral sclerite (SLS) large, occupying almost half of endosoma, strongly broadened basally, constricted medially; the clublike dextrolateral sclerite (DLS) (Fig. 16); and the right paramere sickle-shaped (Fig. 20).

Most similar to *Xenocylapidius gemellus* in sharing the brownish yellow mottling on dorsum (Figs 1, 5), the rounded extreme apex of apical process of left paramere when viewed dorsally (Figs 19, 35), and the sickle-shaped right paramere. This new species can, however, be distinguished by the dark brownish antennal segment and shape of the endosoma (Figs 16).

#### Description.

*Male*. **COLORATION** (Figs 1, 9). Dorsum mostly with mottled, brownish yellow coloration. ***Head*.** Vertex and frons mottled with dark brown and yellow; remainder of head dark red with yellow mottling; antennal segment I dirty yellow, with an indistinct, dark yellow tinge basally and with a reddish tinge occupying apical one third of inner surface; segment II dark brown; labium dark brown with indistinct, dirty yellow areas. ***Thorax*.** *Pronotum*. Collar yellow; calli dark brown, with broad, yellowish mottling; anterior margin weakly tinged with red medially; lateral margin and posterior lobe dark brown, tinged with red and dirty yellow; humeral angle and medial portion of posterior margin yellow. *Mesoscutum and scutellum*. Mostly reddish; mesoscutum weakly tinged with dark brown medially and with dirty yellow area bordering portion being depressed onto lateral margin; scutellum reddish with dirty yellow patch apically. *Thoracic pleura*. Proepimeron and proepisternum mostly dark brown with reddish areas; remaining pleura reddish, with indistinct yellowish areas. *Hemelytron*. Corium and clavus dark brown, mottled with yellow; cuneus dark brown, weakly tinged with red, inner angle yellow, apex with a small, dirty yellow patch; membrane fuscous with indistinct, dirty yellow areas. *Legs*. Procoxa dark brown, dirty yellow apically; meso- and metacoxae yellow; femora dirty yellow brown with reddish areas; tibiae dark brown; tarsi dirty yellow brown. ***Abdomen*.** Dark brown with large dirty yellow areas. **STRUCTURE, TEXTURE, AND VESTITURE** (Figs 1, 9). ***Head*.** Antennal segment II weakly broadened toward apex, covered with moderately dense, adpressed and semirecumbent setae, sparse on basal one-fifth of segment II and dense on remainder of segment. ***Thorax*.** *Pronotum*. Lateral margins sharply carinate, somewhat elevated. *Mesoscutum and scutellum*. Scutellum weakly convex. *Hemelytron*. Covered with short, relatively dense, adpressed, black setae.

***Male genitalia*.** *Aedeagus* (Figs 16–17). Basal sac (BSC) occupying one third of endosoma; sclerotized portion of ductus seminis inside endosoma (DSS) ovoid; secondary gonopore nearly circular, not fully closed; basal fig (BP) irregular in shape; apex of endosoma with two bundles of spiculi (SP1 and SP2); medial sclerite (MS) long, weakly curved, tapering toward apex, sharply pointed; sinistrolateral sclerite (SLS) large, occupying almost half of endosoma, strongly broadened basally, constricted medially, and broadened, nearly cylindrical on apical half; dextrolateral sclerite (DLS) somewhat smaller than SLS, clublike. *Left paramere* (Figs 18–19). Apical process: lateral view: weakly tapering toward apex, obtuse apically; dorsal view: lateral margins weakly curved, extreme apex rounded; paramere body: dorsal view: weakly broadened toward apex; sensory lobe: massive, just slightly tapering toward apex, obtuse. *Right paramere* (Fig. 20). Sickle-shaped; apical process: long, thin, arcuate, just slightly narrowed toward apex; paramere body: thin, dorsal margin straight, ventral margin weakly arcuate.

#### Measurements.

♀/♂ (n=2, holotype measurements second). *Body*. Length 6.00/4.70, width 2.15/1.76. *Head*. Length 1.00/0.98, width 0.85/0.79, interocular distance 0.35/0.35. *Antenna*. Length of segment I 0.74/0.64, II 1.83/1.83 (III and IV missing in both specimens). *Labium*. Length of segment I 0.98/0.95 (II, III, and IV immeasurable in both specimens). *Pronotum*. Length 0.85/0.73, width of anterior margin 0.68/0.65, length of lateral margin 0.98/0.80, width of posterior margin 1.60/1.38.

*Female*. Similar to male in coloration, structure, texture, and vestiture.

#### Biology.

Unknown.

#### Distribution.

**Figure 31.**
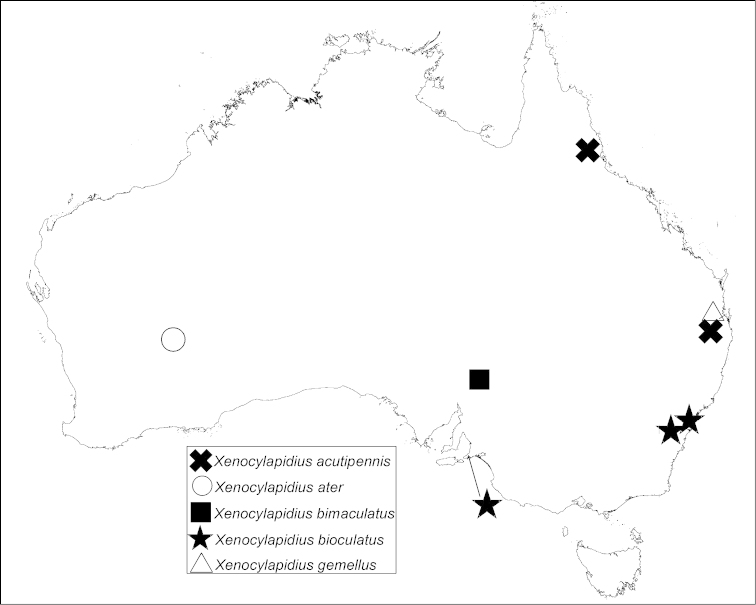
Distribution map of *Xenocylapidius* spp.

Australia (Queensland) (Fig. 31).

#### Etymology.

The specific name is derived from the Latin “acutus”, meaning sharpened, and is used to denote the sharply pointed mesial process (MS) of the endosoma.

#### Type material.

**Holotype** ♂: Malanda; Queensl[and] Mjöberg; Swedish Museum of Natural History Stockholm NHRS (NHRS); paratype 1 ♀: Glen Lamington Queensl[and] Mjöberg; Swedish Museum of Natural History Stockholm NHRS (NHRS).

### Xenocylapidius ater

Taxon classificationAnimaliaHemipteraMiridae

Wolski & Gorczyca sp. n.

http://zoobank.org/DF204786-7FCE-4940-B323-99FA280CCF46

Figures 2 , 10 , 21 –25 , 31

#### Diagnosis.

Recognized by the black dorsal coloration (Fig. 2); the antennal segment II with a yellow annulation apically (Figs 2, 10); the endosomal dextrolateral sclerite (DLS) nearly square on basal one third and triangular on apical two thirds (Fig. 21); the extreme apex of apical process of left paramere when viewed dorsally nearly conelike (Fig. 24); the sensory lobe (SL) of left paramere long, weakly arcuate in dorsal view (Fig. 24); and the right paramere with an apical process broadened with a narrow, nearly conelike process apically (Fig. 25).

Most similar to *Xenocylapidius rolandi* in sharing blackish dorsal coloration (Figs 2, 7). *Xenocylapidius ater* can, however, be easily distinguished by the lack of large white patches on hemelytron (Fig. 2), the coloration of antennal segment II (Figs 2, 10), and the shape of the male genitalia (Figs 21–25).

#### Description.

*Male*. **COLORATION** (Figs 2, 10). Dorsum mostly blackish with small yellow and dirty yellow areas. ***Head*.** Black with yellowish patches; vertex with two yellow patches each situated behind each eye and with additional two longitudinal, yellow patches, each bordering inner margin of each eye, vertex also with a small yellow patch medioapically; frons with two groups of several small, yellowish patches, each situated laterally, near inner margin of eye, frons also with a small, yellow patch medioapically, nearly bordering base of clypeus; clypeus with a short, longitudinal, yellow patch basally; mandibular fig with two small, yellow patches basally, each bordering base of clypeus; mandibular fig also with a yellow line along entire length of ventral margin; ventral surface of maxillary fig and dorsal surface of gula, bordering maxillary fig with a relatively large, yellow patch; gula with a relatively large, yellow patch bordering ventral margin of eye; antenna black except for contrasting yellow annulation at apical one fifth of antennal segment II; labium black with an indistinct, dirty yellowish annulation medially. ***Thorax*.** *Pronotum*. Black with a broad, dirty yellow mottling on pronotal calli. *Mesoscutum and scutellum*. Black. *Thoracic pleura*. Blackish. *Hemelytron*. Blackish; base of embolium with a small, yellow patch. *Legs*. Pro- and mesocoxae black; metacoxa dirty yellow; femora black; mesofemur with a small, dirty yellow patch subapically; metafemur with relatively broad, yellow annulation subapically; tarsi dirty yellow. ***Abdomen*.** Blackish with indistinct, dirty yellowish areas. **STRUCTURE, TEXTURE, AND VESTITURE** (Figs 2, 10). Antennal segment II weakly broadened toward apex, covered with moderately dense, adpressed and semirecumbent setae, sparse on basal one-fifth of segment II and dense on remainder of segment. ***Thorax*.** *Pronotum*. Lateral margins sharply carinate, somewhat elevated. *Mesoscutum and scutellum*. Scutellum weakly convex. *Hemelytron*. Covered with very short, relatively dense, adpressed, black setae.

***Male genitalia*.** *Aedeagus* (Figs 21–22). Basal sac (BSC) occupying one third of endosoma; sclerotized portion of ductus seminis inside endosoma (DSS) stout, with sinuate margins; basal fig (BSC) nearly cylindrical, thin, and sinuate at basal two thirds, nearly rectangular at apical one third; dextrolateral sclerite (DLS) nearly square on basal one third and triangular on apical two thirds. *Left paramere* (Figs 23–24). Apical process: lateral view: nearly cylindrical, weakly constricted medially; dorsal view: weakly tapering toward apex; extreme apex nearly conelike; paramere body: lateral view: dorsal surface covered with dense, long, protruding setae; dorsal view: sensory lobe: long, weakly arcuate. *Right paramere* (Fig. 25). Apical process: broadened with a narrow, nearly conelike process apically; paramere body weakly arcuate, covered with sparse, long, protruding setae.

#### Measurements.

Holotype ♂: *Body*. Length 5.3, width 2.15. *Head*. Length 1.0, width 0.88, interocular distance 0.35. *Antenna*. Length of segment I 0.71, II 1.82, III 0.62, IV (missing). *Labium*. Length of segment I 0.87, II 1.43, III 0.85, IV 0.7. *Pronotum*. Length 0.82, width of anterior margin 0.75, length of lateral margin 1.00, width of posterior margin 1.75.

*Female*. Unknown.

#### Biology.

Unknown.

#### Distribution.

Australia (Western Australia) (Fig. 31).

#### Etymology.

The specific name is derived from the Latin “ater”, meaning black, and is used to denote the blackish dorsal coloration.

#### Type material.

**Holotype** ♂: Australia, WA 06/85, 30 km nnw. Leonora 28.61799S, 121.19967E, 441 m, 30.1.2006, M. Baehr (ZSM).

### Xenocylapidius bimaculatus

Taxon classificationAnimaliaHemipteraMiridae

Wolski & Gorczyca sp. n.

http://zoobank.org/D2ED9060-36D9-4C86-A273-33EB7D120100

Figures 3 , 12 , 26 –31

#### Diagnosis.

Recognized by the chocolate brown dorsum with two large whitish patches, each situated near base of the hemelytron (Fig. 3); the medial sclerite (MS) stout, large, occupying almost half of endosoma, tapering toward apex, sharply pointed (Fig. 26); the extreme apex of apical process of the left paramere weakly arcuate, nearly conelike (Fig. 24); the right paramere with an apical process ovoid, with a basal, small, obtuse process dextrolaterally and paramere body rather thin, nearly cylindrical, and very weakly arcuate at apical half, strongly broadened at basal half (Fig. 30).

Most similar to *Xenocylapidius rolandi* in sharing a large, pale patch near base of hemelytron (Figs 3, 7). The present new species can, however, be distinguished by the chocolate brown dorsum (Fig. 3) and the shape of the male genitalia (Figs 26–30).

#### Description.

*Female*. **COLORATION** (Figs 3, 11). Dorsum chocolate brown, with yellow areas. ***Head*.** Chocolate brown with whitish areas; posterior margin of vertex with two indistinct, dirty yellow patches, each situated mediolaterally, vertex also with two longitudinal, yellowish patches, each bordering inner margin of each eye and with a longitudinal, yellow stripe medially; frons with two yellow patches, each situated laterally and with yellow patch medioapically, bordering clypeus; clypeus with a short, longitudinal, yellow patch basally; mandibular fig with two small, yellow patches basally, each bordering base of clypeus, mandibular fig also with a yellow line along entire length of ventral margin; gula with relatively large, yellow patch bordering ventral margin of eye; antennal segment I chocolate brown with a yellowish annulation near base; segment II dirty yellow to brown, apical one third dark brown; segments III and IV dark brown; labium yellow, with fuscous areas. ***Thorax*.** *Pronotum*. Chocolate brown, with indistinct yellow mottling on anterior half of calli and with indistinct yellow stripe medially, originating from middle of pronotal calli and ending at posterior margin. *Mesoscutum and scutellum*. Chocolate brown with a pale patch apically. *Thoracic pleura*. Chocolate brown. *Hemelytron*. Chocolate brown with indistinct yellowish shades and more or less developed whitish areas; embolium with a small whitish patch basally and apically; corium and embolium with a large, whitish patch near base; cuneus with a small yellow patch apically; membrane chocolate brown, membrane venation whitish. *Legs*. Procoxa chocolate; meso- and metacoxae yellow; profemur chocolate brown; protibia brownish; protarsus dirty yellow. ***Abdomen*.** Brown with yellow areas. **STRUCTURE, TEXTURE, AND VESTITURE** (Figs 3, 11). ***Head*.** Antennal segment II weakly broadened toward apex, covered sparse, adpressed setae, sparse on basal one-fifth of segment II and dense on remainder of segment. ***Thorax*.** *Pronotum*. Lateral margins sharply carinate, somewhat elevated. *Mesoscutum and scutellum*. Scutellum weakly convex. *Hemelytron*. Covered with short, relatively dense, adpressed, black setae.

*Male*. Similar to female in coloration, structure, texture, and vestiture.

***Male genitalia*.** *Aedeagus* (Figs 26–27). Basal sac (BSC) occupying one third of endosoma; apex of endosoma with a single bundle of spiculi (SP1); medial sclerite (MS) stout, large, occupying almost half of endosoma, tapering toward apex, sharply pointed. *Left paramere* (Figs 28–29). Apical process: lateral view: broadened basally, cylindrical at apical two-thirds, obtuse; dorsal view: lateral margins weakly sinuate; extreme apex weakly arcuate, nearly conelike; sensory lobe: smassive, just slightly arcuate, obtuse. *Right paramere* (Fig. 30). Apical process: ovoid, with a basal, small, obtuse process dextrolaterally; paramere body: rather thin, nearly cylindrical, and very weakly arcuate at apical half, strongly broadened at basal half, covered with a few long, protruding setae sinistrolaterally.

#### Measurements.

♀/♂ (n=3, holotype measurements in parentheses). *Body*. Length 4.30–4.70/4.00 (4.70), width 1.65–1.75/1.65 (1.75). *Head*. Length 0.70–0.82/0.88 (0.82), width 0.70–0.73/0.70 (0.73), interocular distance 0.32–0.33/0.30 (0.32). *Antenna*. Length of segment I 0.44–0.50/0.45 (0.50), II 1.20–1.35/1.25 (1.35), III 0.60–0.65/0.63 (0.65) (IV missing in examined specimens). *Labium*. I (holotype) 0.80 (remaining segments immeasurable in examined specimens). *Pronotum*. Length 0.65–0.68/0.65 (0.68), width of anterior margin 0.63–0.65/0.58 (0.65), length of lateral margin 0.73–0.75/0.78 (0.75), width of posterior margin 1.30–1.38/1.33 (1.38).

#### Biology.

Unknown.

#### Distribution.

Australia (South Australia) (Fig. 31).

#### Etymology.

The specific name is derived from the Latin “bi”, meaning two, and “macula”, meaning spot, and is used to denote the presence of two large dorsal patches, each situated near base of each hemelytron.

#### Type material.

Holotype ♀: Australien 78, Wilpena Pound, Flinders Range, SA, 25.12.1972, M. Baehr (ZSM). Paratypes 1 ♀ and 1 ♂: same data as for holotype (ZSM).

### Xenocylapidius bioculatus

Taxon classificationAnimaliaHemipteraMiridae

(Girault)

Figures 4 , 11 , 31

Rhinomiridius bioculatus Girault 1934: l (sp. n.); Carvalho 1957: 24 (catalog), 1974: 43 (list of types of species described by Girault); Cassis and Gross 1995: 150 (list); Schuh 1995: 36 (catalog); Gorczyca and Chérot 1998: 24 (note).
Xenocylapidius australis Gorczyca, 1999: 16, 17, Fig. 2 (sp. n.), (synonymized by Gorczyca 2006) (BPBM).

#### Diagnosis.

Recognized by the following set of characters: dorsum with a mottled, blackish yellow coloration (Fig. 4); apical half of antennal segment II with dense, fine, semirecumbent setae and with sparse, protruding, bristlelike setae; femora entirely blackish (Fig. 4).

Most similar to *Xenocylapidius acutipennis*, *Xenocylapidius gemellus*, *X gressitti*, and *Xenocylapidius tamasi* in sharing mottled dorsal coloration (Figs 1, 4, 5–6, 8). *Xenocylapidius bioculatus* can, however, be distinguished by the presence of bristlelike setae on the antennal segment II and the uniformly black coloration of femora (Fig. 4)

#### Biology.

Unknown.

#### Distribution.

Australia (New South Wales, South Australia) (Fig. 31).

#### Examined material.

Holotype of *Xenocylapidius australis* ♀: Australia N. S. W., Manly nr Sydney, North Head 16-21.2., D. Shcherbakov 1997 (US); 1?: Mt. Gibraltar National Park, N.S.W., 24 Feb 1965, D.K. McAlpine; Eeastern scarp, c. 3000 ft.; Carvalho to Drake Coll 1993 (USNM).

### Xenocylapidius gemellus

Taxon classificationAnimaliaHemipteraMiridae

Wolski & Gorczyca sp. n.

http://zoobank.org/DFE7AE29-2127-47BD-A66F-ED2B6D9822AC

Figures 5 , 31 –36

#### Diagnosis.

**Figures 32–41.**
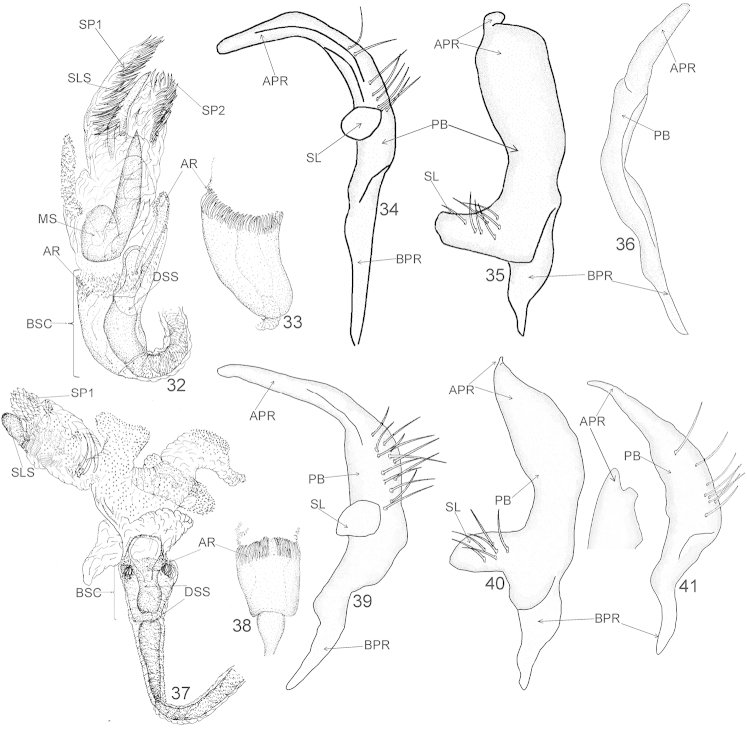
Male genitalia of *Xenocylapidius gemellus* (**32–36**) and *Xenocylapidius rolandi* (**37–41**): **32, 37** Endosoma (dorsal view) **33, 38** Basal sac of endosoma (ventral view) **34, 39** Left paramere (left lateral view) **35, 40** Left paramere (dorsal view) **36, 41** Right paramere (right lateral view). APR = apical process of paramere; AR = apical ring of endosomal basal sac; BPR = basal process of paramere; BSC = basal sac; DSS = sclerotized portion of ductus seminis inside endosoma; MS = medial sclerite; PB = paramere body; SL = sensory lobe; SLS = sinistrolateral sclerite; SP1 and SP2 = endosomal spiculi.

Recognized by the mottled, brownish yellow coloration (Fig. 5); the dirty yellow antennal segment II (Fig. 5); the medial sclerite (MS) stout, occupying more than one third of endosoma, basal one third nearly rounded, apical two thirds tapering toward apex, sharply pointed apically; the endosomal sinistrolateral sclerite (SLS) relatively small, occupying one fourth of endosoma, bifurcate at basal one third, remainder of sclerite cylindrical, somewhat narrowed apically (Fig. 32); the extreme apex of apical process of left paramere rounded in dorsal view (Fig. 35); and the right paramere sickle-shaped (Fig. 36).

Most similar to *Xenocylapidius acutipennis* in sharing a brownish yellow mottling on dorsum (Figs 1, 5), the rounded extreme apex of apical process of the left paramere when viewed dorsally (Figs 19, 35), and sickle-shaped right paramere. This new species can, however, be distinguished by the dark dirty yellow antennal segment (Fig. 5) and the shape of the endosoma (Figs 32).

#### Description.

*Male*. **COLORATION** (Fig. 5). Dorsum dark brown with dirty yellow and whitish areas. ***Head*.** Dark brown dirty yellow; antenna dirty yellow; labium yellowish. ***Thorax*.** *Pronotum*. Dark brown dirty yellow. *Mesoscutum and scutellum*. Dark brown with a whitish patch apically. *Thoracic pleura*. Dark brown with brown and dirty yellow areas. *Hemelytron*. Brown, mottled with yellow; membrane grey, venation dirty yellowish white. *Legs*. Procoxa dark brown; meso- and metacoxa dirty yellowish; pro- and mesofemur dark brownish; remaining segments of pro- and mesoleg dirty yellow. ***Abdomen*.** Dirty yellow. **STRUCTURE, TEXTURE, AND VESTITURE** (Fig. 5). ***Head*.** Antennal segment II weakly broadened toward apex, covered with moderately dense, semirecumbent setae, sparse on basal one-fifth of segment II and dense on remainder of segment. ***Thorax*.** *Pronotum*. Lateral margins sharply carinate, somewhat elevated. *Mesoscutum and scutellum*. Scutellum weakly convex. *Hemelytron*. Covered with short, relatively dense, adpressed, black setae.

***Male genitalia*.** *Aedeagus* (Figs 32–33). Basal sac occupying one third of endosoma, apical ring (AR) extended into long, irregular, apically broadened and serrate sclerite dextrolaterally; sclerotized portion of ductus seminis inside endosoma (DSS) arcuate, nearly cylindrical at basal two-thirds, apically extended into an irregular, nearly ovoid fig; apical one third of endosoma with two bundles of spiculi (SP1 and SP2); medial sclerite (MS) stout, occupying more than one third of endosoma, basal one third nearly rounded, apical two thirds tapering toward apex, sharply pointed apically; sinistrolateral sclerite (SLS) relatively small, occupying one fourth of endosoma, bifurcate at basal one third, remainder of sclerite cylindrical, somewhat narrowed apically. *Left paramere* (Figs 34–35). Apical process: lateral view: broadened and weakly arcuate basally, slightly tapering toward apex, obtuse apically; dorsal view: lateral margins weakly arcuate, extreme apex rounded; sensory lobe: stout, obtuse apically. *Right paramere* (Fig. 36). Sickle-shaped; apical process: relatively long, weakly curved and slightly tapering toward apex; paramere body: thin, arcuate.

#### Measurements.

Holotype ♂: *Body*. Length 5.50, width 2.00. *Head*. Length 0.88, width 0.77, interocular distance 0.33. *Antenna*. Length of segment I 0.75, II 1.8, III 0.75, IV (partly broken). *Labium*. Immeasurable in specimen examined. *Pronotum*. Length 0.83, width of anterior margin 0.68, length of lateral margin 0.90, width of posterior margin 1.70.

*Female*. Unknown.

#### Biology.

Unknown.

#### Distribution.

Australia (Queensland) (Fig. 31).

#### Etymology.

The specific name is derived from the Latin “gemellus”, meaning twin, and is used to denote the similarity of this species to *Xenocylapidius acutipennis*.

#### Type material.

**Holotype** ♂: QUEENSLAND, Cedar Creek, Mars 1910, E. Mjöberg (NHRS).

**Figures 42–46.**
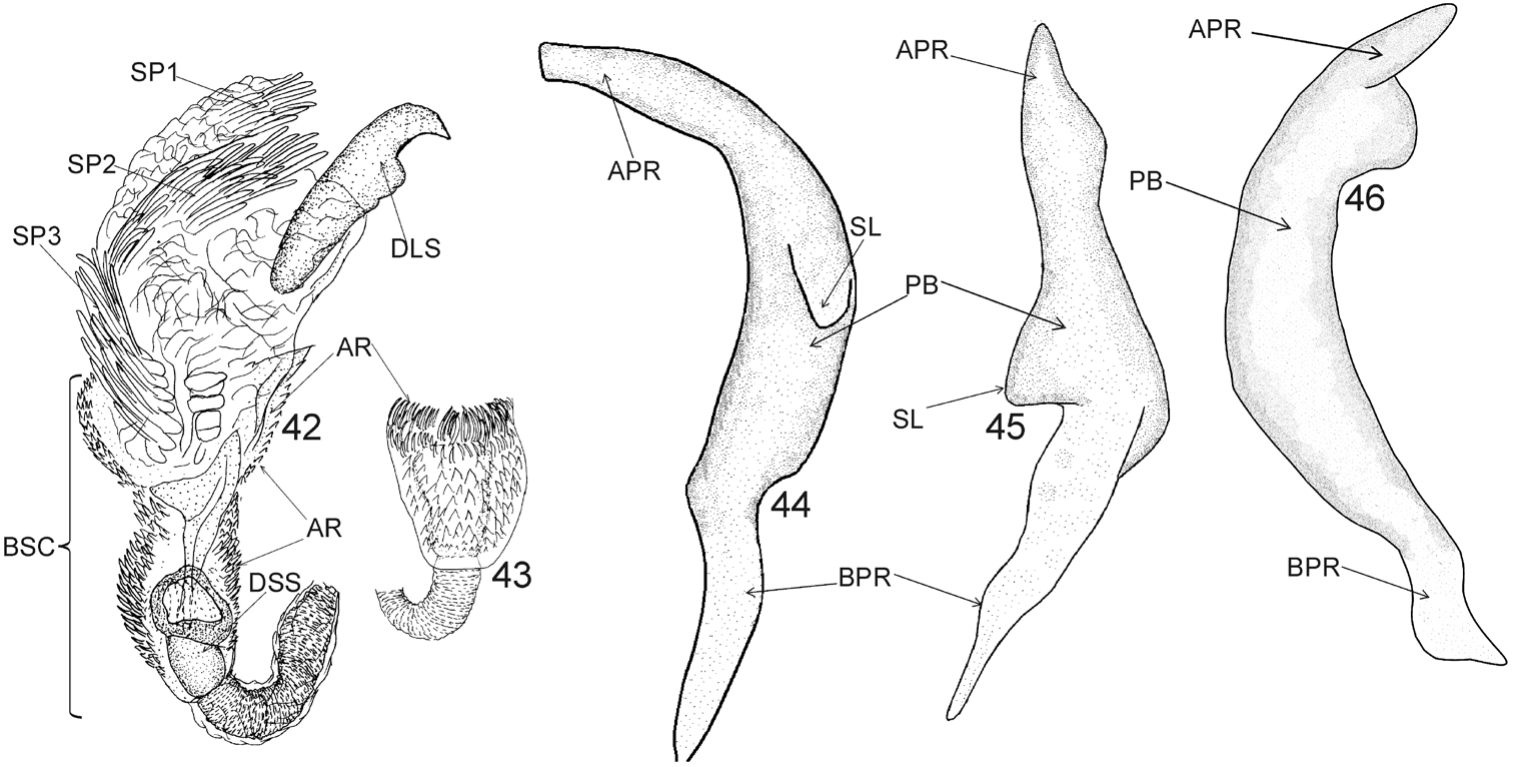
Male genitalia of *Xenocylapidius tamasi*: **42** Endosoma (dorsal view) **43** Basal sac of endosoma (ventral view) **44** Left paramere (left lateral view) **45** Left paramere (dorsal view) **46** Right paramere (right lateral view). APR = apical process of paramere; AR = apical ring of endosomal basal sac; BPR = basal process of paramere; BSC = basal sac; DLS = dextrolateral sclerite; DSS = sclerotized portion of ductus seminis inside endosoma; PB = paramere body; SL = sensory lobe; SP1, SP2, and SP3 = endosomal spiculi.

**Figure 47.**
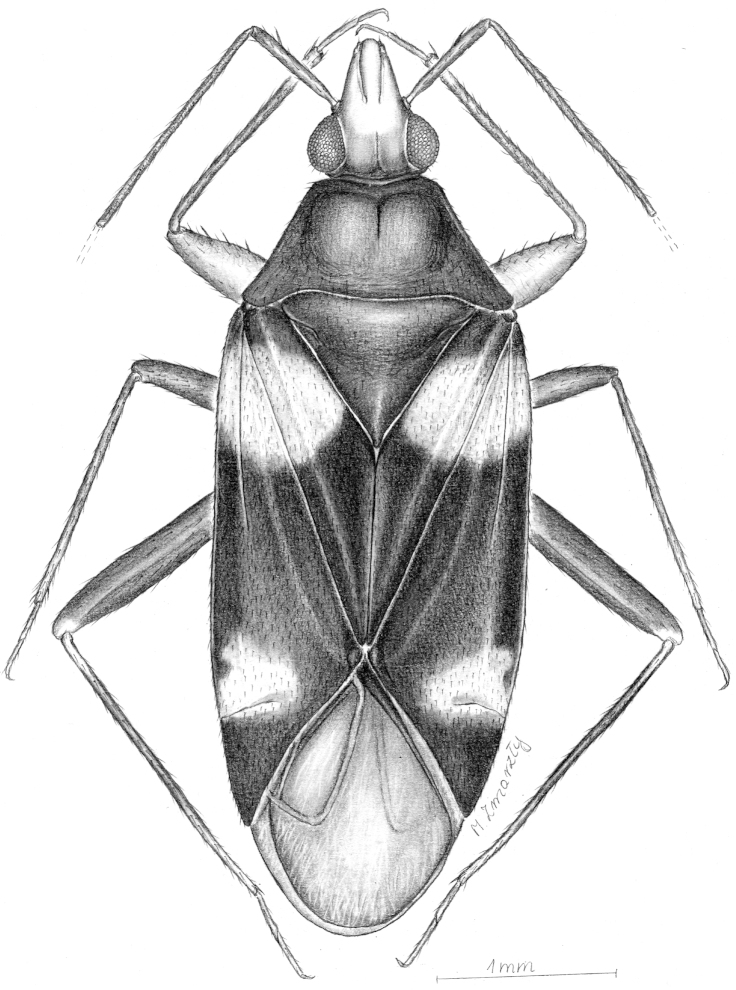
Dorsal habitus drawing of *Xenocylapidius rolandi* (holotype).

### Xenocylapidius gressitti

Taxon classificationAnimaliaHemipteraMiridae

Gorczyca

Figures 6 , 13 , 48

Xenocylapidius gressitti Gorczyca 1999: 16, 19, Fig. 3 (sp. n.), 2006: 70 (catalog).

#### Diagnosis.

Recognized by the following set of characters: dorsum with a mottled, dark brownish yellow coloration (Fig. 6); pronotal collar indistinct; femora entirely blackish, except for pale yellow annulation at basal one third of mesofemur.

Most similar to *Xenocylapidius acutipennis*, *Xenocylapidius bioculatus*, *Xenocylapidius gemellus*, and *Xenocylapidius tamasi* in sharing mottled dorsal coloration (Figs 1, 4, 5–6, 8). *Xenocylapidius gressitti* can, however, be distinguished by the coloration of femora.

#### Biology.

Unknown.

#### Distribution.

**Figure 48.**
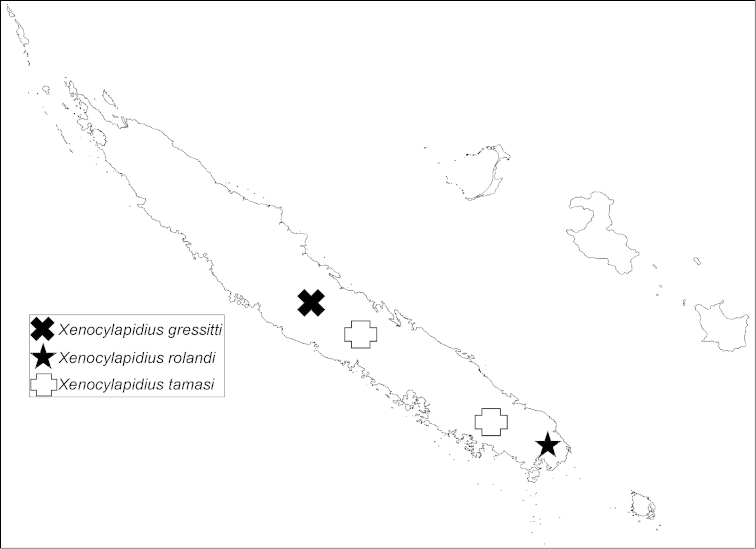
Distribution map of *Xenocylapidius* spp.

New Caledonia (North Province) (Fig. 48).

#### Type material.

Holotype ♀: New Caledonia, Col des Roussettes, 450-550 m, 4–6. II.63; J. L. Gressitt Collector (**BPBM**).

### Xenocylapidius rolandi

Taxon classificationAnimaliaHemipteraMiridae

Wolski & Gorczyca sp. n.

http://zoobank.org/87AF24A7-F7A9-481E-A200-2EEB45EDC679

Figures 7 , 14 , 37 –41 , 47 –48

#### Diagnosis.

Recognized by the white head, with a fuscous vertex (Figs 7, 47); the blackish hemelytron with two large, white patches at base and at apex of corium (Figs 7, 47); the sclerotized portion of ductus seminis (DSS) composed of two parts: basal one, relatively long, gradually broadened toward apex and apical one, weakly ovoid basally and rounded apically; the apical half of endosoma composed of five strongly membranous lobes covered with tiny denticles; the endosomal sinistrolateral sclerite (SLS) small, nearly ovoid, with serrate margins (Fig. 37); and the apical process of right paramere tapering toward apex, with a subapical, short, obtuse process dextrolaterally (Fig. 41).

Most similar to *Xenocylapidius bimaculatus* in sharing large, pale patch near base of hemelytron (Figs 4, 7, 47). The present new species can, however, be distinguished by the blackish dorsum, with a large, white patch situated on hemelytron apically (Fig. 7, 47), and the shape of the male genitalia (Figs 37–41).

#### Description.

*Male*. **COLORATION** (Figs 7, 14, 47). Dorsum blackish with large white areas. ***Head*.** Mostly white; vertex fuscous; frons with two small, fuscous patches, each contiguous with inner margin of each eye and surrounding antennal insertion; gula blackish; antennal segments I and II fuscous; labial segment I blackish; remainder of labium dirty yellow. ***Thorax*.** *Pronotum*. Black. *Mesoscutum and scutellum*. Black. *Thoracic pleura*. Black. *Hemelytron*. Mostly black; corium and clavus with large, white patch near base; apex of embolium, apicolateral surface of corium, and medial portion of inner margin of cuneus with a large white patch; membrane dark grey. *Legs*. Procoxa black; meso- and metacoxae yellow, with a fuscous patch basally; femora and tibiae black; metafemur with a narrow, reddish annulation subapically and yellow, narrow annulation apically; metatibia with a yellow annulation basally and dirty yellow tinge at apical one third; tarsi dirty yellow. ***Abdomen*.** Black. **STRUCTURE, TEXTURE, AND VESTITURE** (Figs 7, 14, 47). ***Head*.** Antennal segment II weakly broadened toward apex, covered with moderately dense, semirecumbent setae, sparse on basal one-fifth of segment II and dense on remainder of segment, apical one fourth also with sparse, bristlelike, protruding setae. ***Thorax*.** *Pronotum*. Lateral margins incarinate, not elevated. *Mesoscutum and scutellum*. Scutellum flattened. *Hemelytron*. Covered with very short, relatively dense, adpressed, black setae.

***Male genitalia*.** *Aedeagus* (Figs 37–38). Basal sac (BSC) nearly square; sclerotized portion of ductus seminis of endosoma (DSS) composed of two parts: basal one, relatively long, gradually broadened toward apex and apical one, weakly ovoid basally and rounded apically; apical half of endosoma composed of five strongly membranous lobes covered with tiny denticles; apical portion of endosoma with a single bundle of short spiculi (SP1); sinistrolateral sclerite (SLS) small, nearly ovoid, with serrate margins. *Left paramere* (Figs 39–40). Apical process: lateral view: slightly tapering toward apex, very weakly curved subapically; dorsal view: strongly tapering toward apex; sensory lobe: stout, obtuse. *Right paramere* (Fig. 41). Apical process: tapering toward apex, thin; dorsal view: tapering toward apex, with subapical, short, obtuse process dextrolaterally; paramere body: dorsal surface with sparse, long, protruding setae.

#### Measurements.

Holotype ♂: *Body*. Length 4.75, width 1.70. *Head*. Length 0.80, width 0.70, interocular distance 0.33. *Antenna*. Length of segment I 0.65, II 1.48 (III and IV missing). *Labium*. Length of segment I 0.87 (II, III, and IV immeasurable). *Pronotum*. Length 0.60, width of anterior margin 0.63, length of lateral margin 0.70, width of posterior margin 1.32.

*Female*. Unknown.

#### Biology.

Unknown.

#### Distribution.

New Caledonia (South Province) (Fig. 48).

#### Etymology.

We are happy to name this species after our friend and colleague and the collector of the type specimen Roland Dobosz (Upper Silesian Museum, Bytom, Poland).

#### Type material.

Holotype ♂: New Caledonia (S), 22°16.8'S, 166°53.5'E, Pic du Grand Kaori, 26. 12. 2006, 240 m, night coll. (lamp & beating), leg. R. Dobosz & M. Wanat; 5915/1788, coll. (MNHN).

### Xenocylapidius tamasi

Taxon classificationAnimaliaHemipteraMiridae

Gorczyca

Figures 8 , 15 , 42 –46 , 48

Xenocylapidius tamasi Gorczyca 1997: 179, Figs l, 3, 6 (sp. n.), 1999: 16, figs 7–9 (redescription, male genitalia), 2006: 70, Fig. 23 (catalog)

#### Diagnosis.

Recognized by the mottled, dark brown, dorsal coloration (Fig. 8), the femora mottled with dark brown and yellow (Figs 8, 15), the endosoma with three bundles of spicules: one situated medially, second subapically, and third apically (Fig. 42), the endosomal basal sac (BSC) occupying half of endosoma, entirely covered with small denticles (Figs 42–43), the endosomal dextrolateral sclerite (DLS) large, occupying nearly one third of endosoma, weakly broadened toward apex, hook-shaped apically (Fig. 42), the sensory lobe (SL) of left paramere short and obtuse in dorsal view (Fig. 45), the right paramere with apical process broadened, with long apical process, weakly tapering toward apex (Fig. 46).

Most similar to *Xenocylapidius acutipennis*, *Xenocylapidius bioculatus*, *Xenocylapidius gemellus*, and *Xenocylapidius gressitti* in sharing mottled dorsal coloration (Figs 1, 4, 5–6, 8). *Xenocylapidius tamasi* can, however, be distinguished by the coloration of femora. From *Xenocylapidius acutipennis* and *Xenocylapidius gemellus* it can be distinguished by the shape of the male genitalia (Figs 42–46).

***Male genitalia*.** *Aedeagus* (Figs 42–43). Basal sac (BSC) occupying half of endosoma, entirely covered with small denticles; endosoma with three bundles of spicules: one situated medially, second subapically, and third apically; dextrolateral sclerite (DLS) large, occupying nearly one third of endosoma, weakly broadened toward apex, hook-shaped apically. *Left paramere* (Figs 44–45). Apical process: lateral view: very weakly broadened at basal two thirds, cylindrical at apical one third, blunt; dorsal view: basal half with sinistrolateral margin weakly convex and dextrolateral margin strongly convex, apical half tapering toward apex; sensory lobe: short and obtuse. *Right paramere* (Fig. 46). Apical process: broadened, with long apical process, weakly tapering toward apex; paramere body: relatively broad, arcuate.

#### Biology.

Unknown.

#### Distribution.

New Caledonia (South Province) (Fig. 48).

#### Type material.

Holotype ♀: New Caledonia, Col d’ Amieu, Ht. Rembtai; 19. I. 1977, leg. J. Balogh; holotype [red label]; *Xenocylapidius tamasi* gen et sp. n., det. J. Gorczyca, 1997 (HNHM).

#### Additional examined material.

2 ♂♂ and 1 ♀: New Caledonia, Mt. des Koghis, 300–600 m, 19. III. 1968; J.L. Gressitt & T.C. Maa Collectors, Bishop Museum; 1 ♀: New Caledonia, Foret di Thi, 29.X. – 1.XI.1967; J. & M. Sedlacek Collectors, Bishop (US).

## Supplementary Material

XML Treatment for Xenocylapidius

XML Treatment for Xenocylapidius acutipennis

XML Treatment for Xenocylapidius ater

XML Treatment for Xenocylapidius bimaculatus

XML Treatment for Xenocylapidius bioculatus

XML Treatment for Xenocylapidius gemellus

XML Treatment for Xenocylapidius gressitti

XML Treatment for Xenocylapidius rolandi

XML Treatment for Xenocylapidius tamasi
